# Quantitative histopathological assessment of ocular surface squamous neoplasia using digital image analysis

**DOI:** 10.3892/ol.2014.2366

**Published:** 2014-07-22

**Authors:** RIMVYDAS AŠOKLIS, AISTĖ KADZIAUSKIENĖ, RASA PAULAVIČIENĖ, DONATAS PETROŠKA, ARVYDAS LAURINAVIČIUS

**Affiliations:** 1 Faculty of Medicine, Vilnius University, Vilnius LT-03101, Lithuania; 2 Centre of Eye Diseases, Vilnius University Hospital Santariškių Klinikos, Vilnius LT-08661, Lithuania; 3 National Center of Pathology, Vilnius University Hospital Santariškių Klinikos, Vilnius LT-08661, Lithuania

**Keywords:** digital image analysis, ocular surface squamous neoplasia, Ki-67

## Abstract

The aim of this retrospective pilot study was to evaluate the Aperio nuclear V9 algorithm as an image analysis tool to observe the histopathological changes of ocular surface squamous neoplasia (OSSN). A histopathological assessment, including the Ki-67 proliferative index (PI) of immunohistochemically-stained tumor conjunctiva (TC) and healthy conjunctiva (HC) tissues, was performed in six cases of OSSN. The Aperio V9 algorithm was applied to digital images of the tissue specimens to count the Ki-67 PI and to measure the nuclear area indices. This digital algorithm was validated using stereological and visual analysis methods. The visual scoring of Ki-67 PI ranged from 22 to 60% (mean, 38.5%), and from 5 to 20% (mean 9.5%) in TC and HC tissue, respectively. The computer-aided analysis, using the Aperio nuclear V9 algorithm, revealed that the Ki-67 PI ranged from 21.5 to 43.5% (mean, 33.6%), and from 1.9 to 21.0% (mean, 11.8%) in the TC and HC tissue, respectively. The stereological method demonstrated that the Ki-67 PI ranged from 30.1 to 51.5% (mean, 41.0%), and from 3.2 to 30.1% (mean, 15.1%) in the TC and HC tissues, respectively. The strongest association in the collinearity of regression analysis was observed between the Aperio nuclear V9 algorithm/stereological models in the TC tissue (r^2^=0.7; P=0.04) and the HC tissue (r^2^=0.7; P=0.03), and the visual/stereological models in the TC tissue (r^2^=0.7; P=0.04) and the visual/Aperio nuclear V9 algorithm models in the HC tissue (r^2^=0.7; P=0.04). A weak and statistically insignificant association was identified between the visual/Aperio nuclear V9 algorithm analysis in the TC tissue (r^2^=0.4; P=0.2) and the visual/stereological models in the HC tissue (r^2^=0.5; P=0.13). No significant difference was observed between the nuclear area of the TC (mean, 36.5 μm^2^) and HC (mean, 35.7 μm^2^; P=0.88) tissues. It was concluded that the Aperio nuclear V9 algorithm is a useful tool for the reliable analysis of histopathological changes of OSSN. The results of this computer-aided algorithm correlate strongly with the stereological method when assessing the Ki-67 PI.

## Introduction

During the last decade, automated diagnostic image analysis methods have been developed to improve the standardization and reproducibility of the results of histopathological evaluation. Immunohistochemistry (IHC) has been of particular interest, as an objective quantitative assessment is often required. Currently, there are different computer-assisted software tools for automatic IHC analysis based on whole-slide imaging, including Aperio software (Aperio Technologies; Vista, CA, USA), an integrated scanning and image analysis system. This tool incorporates positive pixel count nuclear, membrane and cytoplasmic algorithms, therefore, can be employed to measure areas and staining properties (1). Numerous studies have been conducted regarding the employment of Aperio software analysis in clinical practice, as well as in research models (2–4). Objective and reproducible automated analysis has been shown to facilitate diagnosis, prognosis and management of oncologic diseases (5–6). However, to the best of our knowledge, there are no studies concerning automated analysis using the Aperio algorithms in the cases of ocular surface squamous neoplasia (OSSN).

OSSN (including intraepithelial and invasive neoplasia) is a non-pigmented malignant tumor of the conjunctiva that results in significant ocular surface destruction and visual dysfunction. The reported incidence ranges from 0.02 to 3.5 cases/100,000 individuals/year and exhibits an increasing trend in developing countries with a high prevalence of the human immunodeficiency virus (7). OSSN is commonly a slow growing, low-grade malignancy, however, occasionally it acts aggressively and may result in recurrences, invasion, metastases and even mortality, particularly in immunocompromised patients (8). Previous studies described the following prognostic factors for OSSN recurrence: Older age, greater lesion size, tumor invasion, non-radical excision, absence of cryotherapy and an increased level of positive expression of the biomarker, Ki-67 (8–10). Ki-67 is a nuclear antigen, which is expressed in the proliferating cells of healthy and neoplastic tissue, and is evaluated using IHC (11,12). It was hypothesized in the present study that automated image analysis using the Aperio nuclear V9 algorithm may aid with the quantitative assessment of the Ki-67 value in OSSN.

The aim of the present study was to evaluate the Aperio nuclear V9 algorithm as an image analysis tool for the histopathological changes of OSSN. The results of the automated, stereological and visual IHC assessments of tumor conjunctiva (TC) and healthy conjunctiva (HC) tissues were compared. In addition, the nuclear area in the TC and HC cells was quantified. To the best of our knowledge this is the first study to present the results of this algorithm when applied to the analysis of OSSN.

## Materials and methods

### Patients

**Figure 1:**
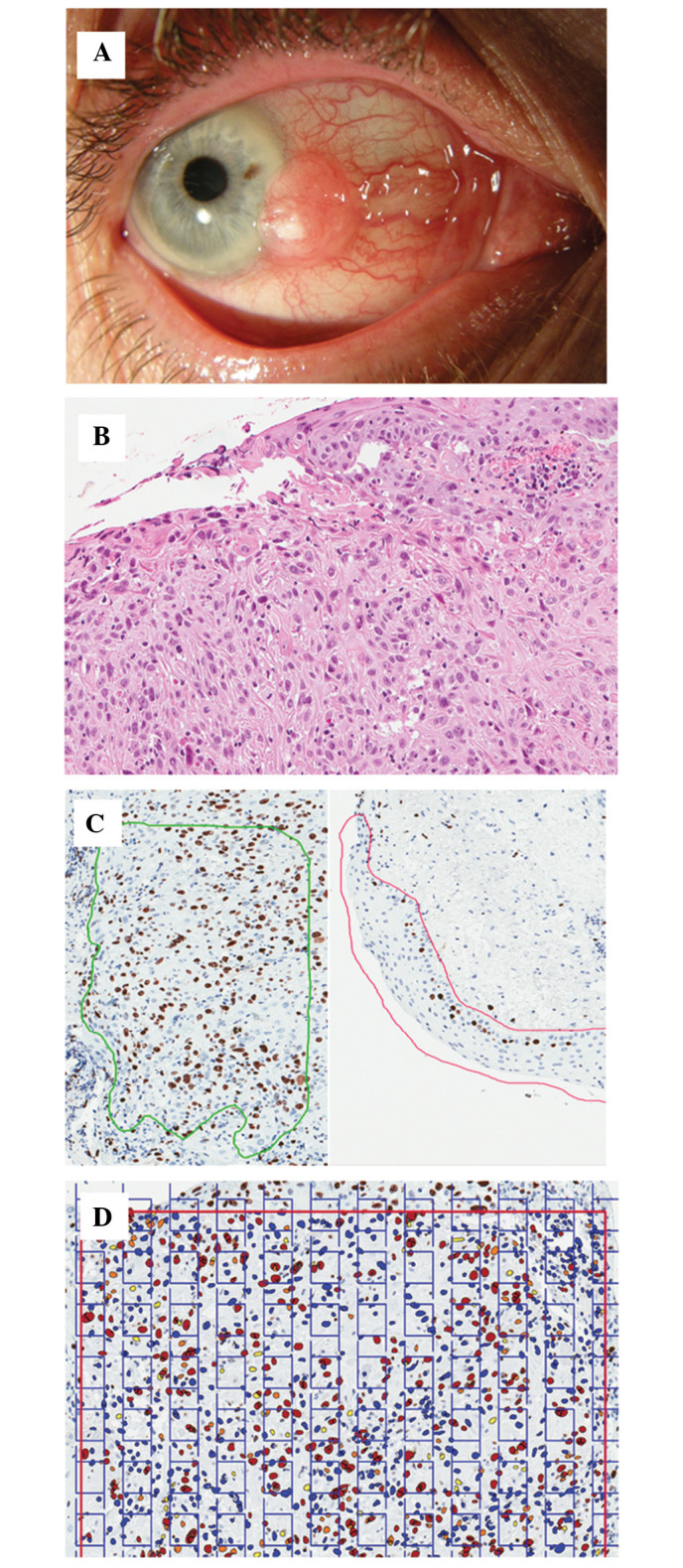
(A) Image of conjunctival squamous cell carcinoma in patient 3 showing a nodular mass with foci of leukoplakia on the surface of the lesion. (B) Digital image of the excisional biopsy (stain, Hematoxylin and Eosin). (C) Ki-67 immunohistochemically-stained slide shows areas of tumor conjunctiva (green) and healthy conjunctiva (red). (B and C: Magnification, ×20). (D) Aperio nuclear v9 algorithm indicates positive nuclei as brown, orange and yellow, and negative nuclei as blue. A grid of the stereology model has been superimposed over the digital Aperio image.

The current retrospective pilot study was conducted at the Centre of Eye Diseases, Vilnius University Hospital Santariškių Klinikos (Vilnius, Lithuania) between April 2002 and June 2012. The study consisted of six conjunctival specimens from six patients with a histopathological diagnosis of OSSN (Fig. 1A and B). In three patients (three eyes) the TC was excised, cryo-application and an amniotic membrane transplantation was performed. In two patients (two eyes) excision of the tumor without any additional surgical manipulation was performed. The data regarding the treatment of one patient is missing. The study was approved by the Ethics Committee of Vilnius University Hospital Santariškių Klinikos, Vilnius, Lithuania (EK-38) Patients provided written informed consent.

### Tissue preparation

Excised tissues for histological analysis were fixed in 10% neutral buffered formalin for 6–24 h and paraffin embedded in a tissue processor (Shandon Pathcentre^®^ Tissue Processor; Thermo Shandon Ltd., Runcorn, UK). Formalin-fixed and paraffin-embedded (FFPE) tissues were sectioned (2-μm thick) and processed for subsequent staining with Hematoxylin and Eosin (H&E). The Ki-67-immunostained slides were prepared according to the manufacturer’s instructions, using the mouse anti-human monoclonal Mib-1 clone antibody (dilution, 1:200; DAKO, Carpinteria, CA, USA) and the Ventana BenchMark XT staining platform (Ventana Medical Systems, Tucson, AZ, USA). Digital images of H&E- and IHC-stained glass slides were obtained using a ScanScope Digital slide scanner (Aperio Technologies) at a magnification of ×20 (Fig. 1B and C).

### Quantification techniques

Various Ki-67 proliferative index (PI) quantification techniques were adopted, including the following: i) Visual evaluation by one pathologist; ii) digital image analysis (DIA) using an LG L226WTQ-SF Flatron monitor (LG Electronics, Seoul, South Korea) and the nuclear V9 algorithm (Aperio ScanScope XT System; Aperio Technologies) counting >1,252 cells in the TC tissue and 242 cells in the HC tissue; and iii) DIA using a stereology module (Stereology Toolkit 4.2.0 [ADCIS, www.adcis.net]; Fig. 1D). Cells exhibiting questionable nuclear staining were discounted (visually and stereologically). Ki-67 PI values from the visual, stereological and automated analyses were compared. In addition, the Aperio nuclear V9 algorithm was used to quantify the area of epithelial nuclei in the TC and HC tissues.

### Statistical analysis

Collinearity in the linear regression analysis was performed with the SAS^®^ 4.2 Enterprise Guide software (SAS Institute Inc., Cary, NC, USA). P<0.05 was considered to indicate a statistically significant difference.

## Results

### Visual, stereological and automated analysis

OSSN was diagnosed in six patients (three males and three females) with a mean age of 69 years (range, 54–84 years). Table I summarizes the clinical, histopathological and management data of these patients. The results of IHC staining for the Ki-67 PI, which was visually assessed by the pathologist, the Aperio nuclear V9 algorithm and using the stereology module are presented in Table II. The visual scoring of Ki-67 PI ranged from 22 to 60% (mean, 38.5%) in TC tissue and from 5 to 20% (mean, 9.5%) in HC tissue. The computer-aided analysis using the Aperio nuclear V9 algorithm demonstrated that the Ki-67 PI ranged from 21.5 to 43.5% (mean, 33.6%) and from 1.9 to 21.0% (mean, 11.8%) in TC and HC tissues, respectively. The stereological method identified a Ki-67 PI range of 30.1 to 51.5% (mean, 41.0%) and from 3.2 to 30.1 (mean, 15.1%) in TC and HC tissues, respectively.

### Collinearity in the regression analysis

The strongest association in collinearity of the regression analysis was observed between the Aperio nuclear V9 algorithm/stereological analysis models in the TC tissue (r^2^=0.7, P=0.04) and in the HC tissue (r^2^=0.7, P=0.03), as well as in the visual/stereological models in the TC tissue (r^2^=0.7, P=0.04) and visual/Aperio nuclear V9 algorithm in the HC tissue (r^2^=0.7, P=0.04). A weak and statistically insignificant association was identified between the Aperio nuclear V9 algorithm/visual analysis in the TC tissue and the visual/stereological analysis in the HC tissue; r^2^=0.4 (P=0.2) and r^2^=0.5 (P=0.13), respectively. No significant difference was observed between the nuclear area of the TC tissue (mean, 36.5 μm^2^ and range, 33.6–38.2 μm^2^) and the HC tissue (mean, 35.7 μm^2^ and range, 33.0–40.0 μm^2^; P=0.88) (Table III).

## Discussion

In the last decade, progress in information and communication technologies has offered novel possibilities for increasing the level of objectiveness and effectiveness of histopathological examination using automated image analysis. However, until recently, the insufficient quality obtained during image acquisition, and of program software, prevented the routine use of automated histopathological analysis in the clinical and research settings. As a result of technological improvements, virtual microscopy and DIA are currently widely available, and there are numerous studies regarding the diagnostic and prognostic value of computer-assisted histopathological analysis (2,3,5,1,13,14). In the present study the experience of adopting automated histopathological analysis for the analysis of OSSN is described and, to the best of our knowledge, this is the first study to apply this analysis method to OSSN.

There are various solutions available for computer-assisted image analysis, ranging from general-purpose software to fully- or semi-automated commercial packages (15). All of these systems aim to achieve an accurate, reproducible, increasingly efficient and economical method of histopathological diagnosis. It is particularly important in quantitative analysis where variability and subjectivity significantly influences the accuracy. Therefore, the different methods of evaluating the IHC staining intensity, including direct observation under a microscope, stereological analysis (by superimposing grids on images) and computer-aided analysis, are often compared. In the present study, the strongest association in regression analysis was observed between the results of digital evaluation using the Aperio nuclear V9 algorithm and the stereological method, with the latter considered to be the gold standard (an independent variable in statistical analysis). The association between the results of automated DIA and the visual method in the HC tissue revealed a similar r^2^-value, however, was identified to be weaker in the TC tissue. This may result from a more regular arrangement of cells (thus facilitating cell counting), and fewer lymphocytes and reactive stromal fibroblasts (which could be mistaken for tumorous cells) in the HC tissue, when using automated analysis. Previous studies have reported a good correlation between the results of visual IHC and automated analysis using Aperio nuclear V9 algorithms, and concluded that the latter method is reliable in histopathological examination (3,13,14,16,17).

Visual analysis is considered to be a subjective, and time- and human resource-consuming process; its weakest point being inter- and intra-observer variability. Differences in the competency of pathologists, and preconceptions, expectations and fatigue are considered to be the primary causes of the abovementioned disadvantages and may lead to inaccurate results and erroneous managerial decisions (15). By contrast, automated image analysis offers a more rapid and effective approach and demonstrates improved reproducibility (14,16). The elimination of subjectivity risk is another advantage of automated analysis, which aids with obtaining increasingly objective qualitative and, importantly, quantitative results. In addition, automated image analysis simplifies the collection, sharing and visualization of data. However, the limitations of computer-assisted image analysis are artifacts in histopathology, exceptional variations in the sample and non-uniform staining, which result in significant issues with the accuracy of the results. Thus, certain studies emphasize the necessity of standardization, strict quality control of sample slides and supervision by a pathologist in order to obtain precise and accurate results (1,2).

The use of the Aperio nuclear V9 algorithm for quantification of the expression level of various biomarkers is considered to be valuable in numerous types of malignancy. Automated Aperio analysis of estrogen and progesterone expression in breast cancer was identified to be valuable for prognostic and therapeutic strategies in previous studies (2,5). Chabot-Richards *et al* (3) demonstrated a significant correlation between the results estimated by a pathologist and those obtained using automated image analysis of the percentage of Ki-67 positivity in diffuse large B-cell lymphoma. Singh *et al* (6) adopted the Aperio analysis method and demonstrated that ovarian cancer cells express chemokine receptor, CCR9 and its ligand, CCL25 and indicated that this chemokine-receptor axis may be involved in ovarian cancer progression.

The Ki-67 proliferation biomarker is widely used in histopathology to measure the population of actively cycling cells, and has been identified as a significant index for the diagnosis and prognosis of different types of malignancy (18–20). Certain studies reported Ki-67 to be an independent prognostic marker for conjunctival squamous cell carcinoma (9,21). The percentage of Ki-67 positivity, as assessed by the Aperio nuclear V9 algorithm in the current study, was comparable with the data obtained by Jung *et al* (11). McKelvie *et al* (9) reported smaller values of Ki-67 staining as a result of visual analysis. However, an association between the Ki-67 positivity and the disease outcome was not determined due to the limited sample size.

According to Wolberg *et al* (22) the nuclear area of the cells of benign breast masses is smaller compared with that of malignant masses. Therefore, in the present study, it was hypothesized that the nuclear area of HC cells may be smaller than that of the TC cells of OSSN. However no significant difference was identified between the nuclear area of the TC and HC cells.

In conclusion, the Aperio nuclear V9 algorithm is considered to be a useful tool for the reliable analysis of histopathological changes of OSSN. The results of this type of DIA algorithm correlate strongly with the stereoscopic method when assessing the Ki-67 PI and may facilitate the accurate quantitative evaluation of IHC-stained biomarkers. Future studies are required to further evaluate the potential of the Aperio nuclear V9 algorithm for the analysis of OSSN.

## Figures and Tables

**Table I:** Clinical, histopathological and management data of the patients exhibiting ocular surface squamous neoplasia.

Patient	Age at diagnosis (years)/gender	Histopathological diagnosis	Location of the tumor	Management	Excisional margins
1	60/Female	Keratinizing invasive CSCC (pT1)	Nasal bulbar, conjunctiva limbus	Excision	No data due to poor orientation of the specimen
2	74/Female	Non-keratinizing invasive CSCC (≥pT1)	Nasal bulbar conjunctiva, limbus	Excision	Involved
3	54/Male	Non-keratinizing invasive CSCC (≥pT1)	Nasal bulbar conjunctiva, limbus	Excision + Cryo + AMT	Clear
4	76/Female	Keratinizing invasive CSCC (pT2)	Nasal bulbar conjunctiva, limbus	Excision + Cryo + AMT	Involved
5	66/Male	Invasive CSCC (pT2)	No data	No data	No data
6	84/Male	Keratinizing CSCC *in situ* (CIN 3)	Temporal bulbar conjunctiva, limbus, cornea	Excision + Cryo + AMT	Clear

pT1, pathological conjunctiva carcinoma stage 1 (tumor ≤5 mm in greatest diameter); pT2, pathological conjunctiva carcinoma stage 2 (tumor >5 mm in greatest diameter, without invasion of adjacent structures). CSCC, conjunctival squamous cell carcinoma; Cryo, cryotherapy; AMT, amniotic membrane transplantation; CIN, conjunctival intraepithelial neoplasia; CIN 3, conjunctival intraepithelial neoplasia grade 3.

**Table II:** Results of visual, automated (Aperio nuclear V9 algorithm) and stereological analysis of the Ki-67 PI.

	Ki-67 PI of tumor conjunctiva (%)			Ki-67 PI of healthy conjunctiva (%)		
		
Patient	Visual	Aperio V9	Stereological	Visual	Aperio V9	Stereological
1	34	34.0	42.0	7	13.2	22.8
2	40	33.4	45.7	7	13.6	15.7
3	60	41.0	51.5	20	21.0	30.1
4	22	28.0	30.1	6	7.2	9.7
5	35	21.5	30.9	5	1.9	3.2
6	40	43.5	45.8	12	14.0	9.0
Mean	38.5	33.6	41.0	9.5	11.8	15.1

PI, proliferative index.

**Table III:** Mean nuclear area of the tumor and healthy conjunctiva as assessed using the Aperio nuclear V9 algorithm.

	Mean nuclear area (μm^2^)	
	
Patient	Tumor conjunctiva	Healthy conjuctiva
1	38.2	34.7
2	33.6	36.7
3	35.3	33.0
4	36.7	34.1
5	38.0	35.4
6	37.4	40.0
Mean	36.5	35.7
